# Tennessee State Medical Society

**Published:** 1891-05

**Authors:** 


### TENNESSEE STATE MEDICAL SOCIETY.

FIFTY-EIGHTH ANNUAL MEETING HELD IN NASHVILLE, APRIL 14, 15, AND 16, 1891.

FIRST DAY—*Morning Session.*

The Society met at Watkins' Hall, and was called to order by the President, Dr. George A. Baxter, of Chattanooga, at 10:30 A. M.

Prayer was offered by Rev. C. D. Elliott, of Nashville.

Dr. T. J. Happel, of Trenton, read a paper entitled "Abscesses." He said the field is a profitable one for thought and investigation, especially in the direction of diagnosis. So far as the treatment is concerned the Latin expression, *ubi pus, ibi incisio*, gives us the therapy of such cases in a few words, so far as their last stages are concerned.

The prophylactic treatment is a different matter. Everything that can be done to prevent pus, to prevent the development of an abscess, must be resorted to, but when pus is present the knife is the instrument for relief. An aspirator can remove the pus itself, but the cause of it, the pyogenetic something is left behind. A free outlet must be given to the pus, the cavity carefully cleaned, perfect draininge secured, arrangements made for thoroughly flushing the diseased organ with antiseptic fluids, the strength of the patient maintained by a generous diet, and nature aided by an abundance of pure fresh air to repair the broken down constitution.

Dr. Happel reported a case of abscess of the spleen. This was a rare trouble, many of our best authorities never having met with a single case. He had in the course of seventeen years' practice found two cases, one due to pressure upon the organ, and the other to chronic malarial poisoning. He also reported a case of abscess of the cornea forming hypopyon, and one case of abscess of the liver which came under his observation recently. In closing, he called attention to the peroxide of hydrogen as one of the best, if not the best, of all agents, used to cleanse and restore to a normal state all pyogenic membranes, surfaces and cavities. As one writer expressed it: "It hunts out pus in all its ramifications as a ferret does a rat."

---

FIRST DAY.—*Afternoon Session.*

Dr. George R. West, of Chattanooga, contributed a paper on "Ovulation and Menstruation."

Individual opinions and theories are as those who love darkness rather than light and insist upon remaining in darkness rather than to be disturbed by the entrance of facts which might bring light. The subject of ovulation and menstruation, their dependence or independence, is one of these benighted fields where individual opinions and theories run riot, and where the light of facts gained from research and experience is so perverted as to render uncertain the supposed certainty that has previously existed.

After giving a *resume* of the literature on the subject, Dr. West drew the following conclusions:

1. That the increased familiarity with the pelvic organs, the result of modern surgery has not materially added to our knowledge of their functions.

2. That though the ovular theory of menstruation has not been overthrown, yet the weight of accumulating evidence seems against it.

3. That the most recent observations point to a common nervous origin for both ovulation and menstruation, and yet an individual independence.

Dr. Thomas M. Woodson, of Gallatin, followed with a paper entitled:

TREATMENT OF PNEUMONIA—THE PAST AND PRESENT METHODS—HAS THE RATE OF MORTALITY BEEN CHANGED?

He briefly reviewed the literature on pneumonia to illustrate the opinions of medical teachers and authors. He was glad that Hare, of Philadelphia, in his work on "Practical Therapeutics," revived the old lines of treatment. He extolled veratrum viride, and said that in the first stage of the disease it is very useful as a medicant. Its two alkaloids possess different influences, and that between them they fulfill every object that is sought after. *Jervine*, a powerful vaso-motor depressant, relaxes the walls of the blood vessels everywhere, at the same time it quiets the action of the heart by an action over its muscle or ganglia as to reduce its force, thus preventing engorgement of the lung; while *veratroidine,* by stimulating the inhibitory nerves of the heart, also slows its beat, fills the ventricles and allays excitement. The advantages of veratrum viride are its completeness and rapidity of action; the fact that it preserves in healthy blood-vessels the blood which may be needed in the crisis, if the disease is not aborted; and its safety is a point largely in its favor. He said that in the second stage, to prevent heart failure by engorgement from over-distension, Dr. Hare gives digitalis with strychnine to stimulate the respiratory centers; that he thinks alcohol in the second stage is inferior to digitalis; carbonate and muriate of ammonia are valuable adjuncts in the second and third stages. He uses opium sparingly for troublesome cough in the later stages, and not in the first stage.

In the first stage of croupous pneumonia, the indications are clear:

1. To control the circulation and diminish the determination of blood to the lungs.
2. To reduce the temperature, if high.
3. Allay pain by rest, physical and physiological.
4. Support the vital powers.

The first two indications are met by veratrum viride better and with more certainty than any other. The third, to allay pain, we have but one remedy—opium or its salts, which stands without a rival. Fourth, to support the patient with especial reference to failing heart and respiratory centers; digitalis, strychnine and alcohol for the latter stages.

More than twenty years ago the speaker expressed the opinion that in inflammatory affections, veratrum viride was a sedative of the greatest value, controlling the action of the heart as effectually as blood-letting, without the exhaustion that must follow the loss of blood. Arterial excitement is reduced by it, while the vital forces are economized. It is especially adapted in pneumonia in the stage of engorgement, in which it appears to bring about prompt resolution. It may be used in the treatment of children with safety. Its constitutional effects having been secured, there is a reduced force and frequency of the circulation, reduction of temperature and respiration, and an amelioration of all the symptoms of the disease. While extolling the virtue of veratrum viride, he would not rely on it alone in pneumonia, as opium was unquestionably entitled to a prominent place, palliative and curative in its action, allaying pain, cough and nervous irritation, available in the latter as well as the early stages.

PHTHISIS PULMONALIS, WITH ESPECIAL REFERENCE TO PROPHYLAXIS.

This was the title of a paper read by Dr. J. R. Buist, of Nashville.

As physicians, impressed with the claims of suffering humanity, we should never relax our efforts as long as consumption, with its multiplied ills, afflicts our race, with its sickness, pain and death. Nor have we any right, as scientists, to despair of the ultimate triumphs of knowledge and the practical results of scientific research. The acknowledged failure of all the proposed plans for the cure of phthisis, based upon therapeutical agents, should lead us upon other lines of effort for its destruction. The impossibility of procuring for the mass of consumptives the benefits of climate and altitude, even if these benefits approximated the value some assign them, should also admonish us to look to the higher plane of preventive medicine in dealing with this disease.

Regarding Koch's tuberculin or parataloid as a remedy for consumption, Dr. Buist said the high expectations so recently excited in these innoculations do not seem to be verified. Certainly for advanced stages of phthisis, and many other conditions of tuberculosis it is unsuited, and positively dangerous; and it is not settled whether any benefit can attend its use in the incipient cases. In making this statement, he would not detract anything from the real value and merit of the discovery, and meant no disparagement of the genius of Koch.

Preventive medicine is, after all, the acknowledged aim and end of scientific research. Though still in its infancy, it has accomplished wonders for humanity. And it is obvious that its first and highest triumphs are to be won among the class of zymotic and infectious maladies. The power of prevention is incalculably more precious than any therapeutical measures. It is therefore highly incumbent upon us individually and collectively, first, to assure ourselves of the modern theory of consumption, and so convinced, we should direct our attention and efforts to a rational prophylactic of this fatal disease.

It may be said that the true difficulty is to get the public to realize its danger from various sources, and still more, to have wise prophylactic measures adopted. This is in the main, true; yet education can perform wonders. The benefits of sanitary reform are now acknowledged and trusted, although fifteen years ago it met with indifference and opposition. It was thought to be a direct attack upon the rights of the citizen. When first inaugurated in Nashville it encountered opposition from every quarter.

The speaker closed with the words of Dr. E. O. Shakspeare, of Philadelphia: "What can and should be done to limit the prevalence of tuberculosis in man? What use was it for Koch to have made his discovery of the infectious nature of the bacillus tuberculosis, if the practitioners of medicine, those who come in direct contact with the people, who are the natural agents for arousing such a public sentiment and enforcement of laws for the protection of public health; utterly neglect to act upon the ample and exact knowledge which we possess concerning the etiology and prophylaxis of tuberculosis."

---

### FIRST DAY—*Evening Session.*

The public were invited to attend this session. Addresses were delivered by Hon. William Litterer, Mayor of Nashville; Hon. H. H. Norman, and Judge J. M. Dickinson.

Dr. Geo. A. Baxter also delivered the President's address, taking for his subject, "Topics of Import to the Profession and Public." The address was scholarly, very instructive, and was listened to with marked attention.

[*Concluded in Next Issue.*]

